# Endosymbiont Tolerance and Control within Insect Hosts

**DOI:** 10.3390/insects3020553

**Published:** 2012-06-15

**Authors:** Carolin Ratzka, Roy Gross, Heike Feldhaar

**Affiliations:** 1 Department of Microbiology, Biocentre, University of Würzburg, 97074, Germany; E-Mails: carolin.ratzka@biozentrum.uni-wuerzburg.de (C.R.); roy@biozentrum.uni-wuerzburg.de (R.G.); 2 Animal Ecology I, University of Bayreuth, 95440, Germany

**Keywords:** insects, immune response, endosymbiosis

## Abstract

Bacterial endosymbioses are very common in insects and can range from obligate to facultative as well as from mutualistic to pathogenic associations. Several recent studies provide new insight into how endosymbionts manage to establish chronic infections of their hosts without being eliminated by the host immune system. Endosymbiont tolerance may be achieved either by specific bacterial adaptations or by host measurements shielding bacteria from innate defense mechanisms. Nevertheless, insect hosts also need to sustain control mechanisms to prevent endosymbionts from unregulated proliferation. Emerging evidence indicates that in some cases the mutual adaptations of the two organisms may have led to the integration of the endosymbionts as a part of the host immune system. In fact, endosymbionts may provide protective traits against pathogens and predators and may even be required for the proper development of the host immune system during host ontogeny. This review gives an overview of current knowledge of molecular mechanisms ensuring maintenance of chronic infections with mutualistic endosymbionts and the impact of endosymbionts on host immune competence.

## 1. Introduction

Insects are the most diverse and successful animal group on earth, which is reflected in the variety of habitats they live in, their abundance and species richness. The successful occupation of a large variety of ecological niches is often facilitated by symbiotic microorganisms. Bacterial endosymbionts have been shown to confer various fitness advantages on their insect hosts such as nutritional upgrading [1,2,3], thermal tolerance [4] or enhanced pathogen/parasitoid resistance [5,6,7,8,9,10,11,12]. Bacterial endosymbionts of insects can be classified into primary and secondary symbionts. Primary endosymbionts have an obligate and ancient association (up to estimated 250 million years) with their insect host [13]. Their transmission to the host progeny occurs mainly vertically via the germ line. These symbionts frequently reside in specialized host cells (so-called bacteriocytes) that may form an organ called the bacteriome [14]. In contrast, secondary endosymbionts are usually facultative symbionts, that are not restricted to bacteriocytes, but may also be found extra- or intracellularly in other host tissues, as well as in the haemocoel [15]. They are generally maternally transmitted, but horizontal transmission is also possible [7,15,16]. In contrast to primary endosymbionts, secondary endosymbionts are not required for host development and are usually not carried by all individuals of a host species.

Strikingly, endosymbionts are maintained within their insect hosts in spite of the broad spectrum of defense mechanisms against microbial intruders [17]. The insect innate immune system consists of cellular and humoral components. The cellular immune response comprises the activity of insect haemolymph cells (so-called haemocytes) against pathogens, involving phagocytosis and encapsulation. The humoral immune response includes the production and release of antimicrobial peptides (AMPs), as well as melanization and clotting processes [17,18]. These defense reactions are triggered by the recognition of microbe-associated molecular patterns (MAMPs). The recognition of non-self is facilitated by pattern recognition receptors (PRRs), which specifically bind different MAMPs. Insects have various PRRs for the recognition of different types of microorganisms. Peptidoglycan-recognition proteins (PGRPs) can specifically bind lysine-type peptidoglycan (found in almost all Gram-positive bacteria) or diaminopimelic acid-type (DAP-type) peptidoglycan (typical for Gram-negative bacteria) [19,20]. The other family of PRRs are Gram-negative binding proteins (GNBPs), which were shown to bind to bacterial ligands such as lipopolysaccharides, lipoteichoic acids, or fungal β-1, 3-glucans [21,22,23]. In *Drosophila*, MAMP recognition mainly leads to signal production via the pathways Toll, Imd (immune deficiency), JAK/STAT (janus kinase/signal transduction and activator of transcription) and/or JNK (c-jun N-terminal kinase) and results in the production of AMPs with activity against different types of microbes [17,24,25]. 

At first glance, it seems surprising that bacterial endosymbionts are not eradicated by the host immune system, as they are often very closely related to pathogenic bacteria [26], which are recognized and fought by the insect immune system. This raises the question, which mechanisms allow insect hosts to maintain a chronic infection with bacterial symbionts. Are bacterial endosymbionts not recognized as non-self by the insect immune system or just hidden from it via compartmentalization? Do they actively manipulate and/or overcome the immune response? Recently, several studies have addressed these questions in different model organisms. In this review, we will give an overview of current knowledge of the mechanisms leading to endosymbiont tolerance and control in insects.

## 2. Tolerance Mechanisms

### 2.1. Bacterial Adaptations

Some bacterial endosymbionts seem to have special adaptions allowing them to avoid or resist the host immune response. Evasion of the insect´s immune system can be attained by altered surface structures used for pattern recognition. Thus, polymorphisms in outer membrane proteins seem to contribute to host tolerance of symbiotic bacteria. A prominent example is *Sodalis glossinidius*, a secondary facultative endosymbiont of the tsetse fly *Glossina morsitans morsitans*. *Sodalis* occurs in the gut, haemolymph and even within phagocytic haemocytes of its tsetse host [27]. Weiss *et al*. (2008) found amino acid substitutions and insertions in the exposed loop domains of the *Sodalis* major outer membrane protein (OmpA), which were absent in OmpA proteins from related bacteria pathogenic for tsetse flies such as *E. coli* K12. Infections of tsetse flies with *E. coli* K12 are usually lethal. However, flies survived exposure to a recombinant *E. coli* strain expressing *Sodalis* OmpA instead of its own. Conversely, a recombinant *Sodalis* strain expressing *E. coli* OmpA became virulent. Gene expression profiling revealed that virulent bacteria (expressing *E. coli* OmpA) hardly elicited an induction of immune genes, such as the antimicrobial peptide cecropin. In contrast, avirulent bacteria (expressing *Sodalis* OmpA) triggered a strong immune response [28]. This counterintuitive result of only a weak immune reaction towards virulent bacteria was probably caused by the strong induction of the *pgrp-lb* gene, encoding a known negative immune regulator in *Drosophila* [29]. Interestingly, the recombinant *E. coli* expressing *Sodalis* OmpA was eliminated by the host immune response, whereas wildtype *Sodalis* survived. This is probably due to the fact that *Sodalis* is resistant to several host AMPs [30,31]. It was argued that *Sodalis*, by invading host haemocytes via a type three secretion system (T3SS) [28,32], may also escape elimination by encapsulation or melanization processes.

In contrast to *Sodalis*, which seems to tolerate the host immune response, other bacterial endosymbionts, such as *Wolbachia* [33] and *Spiroplasma* [34] may either not elicit an immune response or may actively interfere with the host immune system, thus preventing an immune response. *Wolbachia* are a group of obligate intracellular and maternally transmitted bacteria, which are extremely widespread in arthropods and are presumably found in up to 66% of all insects [35]. The secret of the success of this bacterium is its ability to manipulate host reproduction by inducing cytoplasmic incompatibility, parthenogenesis, feminization and male-killing [36,37,38]. It was shown that *Wolbachia* does not trigger the expression of the AMPs diptericin, cecropin and defensin in the *Drosophila simulans* Riverside (DSR) strain [33]. However these *Wolbachia*-infected *D. simulans* lines were still able to mount an immune response after challenge with *E. coli*. Similar results were obtained after immune challenge of single and double *Wolbachia*-infected *Aedes albopictus* lines. Accordingly, it was suggested that *Wolbachia* neither induces nor suppresses AMP expression [33]. A microarray analysis using *Wolbachia*-infected *Drosophila* cells confirmed these results for the AMPs diptericin, cecropin and defensin, but also revealed that several other genes involved in Toll and IMD immune signaling were upregulated [39]. Furthermore, recent studies indicated that the interactions between *Wolbachia* and its host are highly specific for combinations of *Wolbachia*-strain and host, as *Wolbachia* for example strongly induced immune gene expression in *Aedes aegypti* [40] and *Anopheles gambiae* [41]. A transcriptomic approach in the parasitoid wasp *Asobara tabida* comparing gene expression in two different wasp strains (Pi3 and NA) with and without symbionts revealed that the Pi3 host strain generally showed a more pronounced immune response towards *Wolbachia* than the NA strain [42]. The immune genes induced in symbiotic wasps were mainly located upstream in the immune pathways, whereas downstream effectors, such as AMPs, were rather downregulated [42]. This finding highlighted the fact that *Wolbachia* is indeed detected by the host organism, but subsequently extensively modifies the host immune response to escape elimination. The mechanisms of how *Wolbachia* interferes with the host immune system have not yet been well characterized. The *Wolbachia* genome sequence revealed the presence of a functional type four secretion system (T4SS) and an unusually high number of genes encoding ankyrin repeat (ANK) proteins [43]. ANKs are known to mediate protein-protein interactions [44] and the T4SS may be used to export ANK effector proteins to the host cytoplasm [45], likewise to intracellular pathogenic bacteria [46]. Interestingly, it was shown that polydnaviruses use ANK proteins to suppress the insect immune system [47].

*Spiroplasma* endosymbionts of insects can be found both extra- and intracellularly and can form a range from mutualistic to pathogenic associations with their hosts [48]. *S. poulsonii* is associated with *Drosophila*, where it colonizes different host tissues, but is predominantly found in the haemolymph [49]. *D. melanogaster* is naturally infected by the male-killing MSRO (melanogaster sex ratio organism) *Spiroplasma* strain, which was only recently discovered [50]. The NSRO (nebulosa sex ratio organism) *Spiroplasma* strain was isolated earlier and transferred from *D. nebulosa* to *D. melanogaster* [51]. It was shown that infection of *D. melanogaster* with MSRO or NSRO *Spiroplasma* strain did not induce the expression of AMPs [34,52] or other immunity-related genes [53]. Furthermore, infected hosts were still able to induce immune gene expression after septic injury. The lack of immune recognition may be due to the fact that *Spiroplasma* are wall-less bacteria [54] and therefore lack MAMPs like peptidoglycan that usually trigger insect immune responses [55]. A recent study on a male-killing NRSO *Spiroplasma* strain, however, demonstrated that the bacterium actively interferes with the host immune system as it was capable to suppress constitutive expression of some immune genes in immune-unchallenged insects [56]. The susceptibility of *Spiroplasma* to *D. melanogaster *immune response seems to be *Spiroplasma*-strain specific. In mutant flies with constitutively activated Toll pathway the NSRO titer was significantly decreased [34], whereas ectopic activation of Toll or Imd pathway led to an increase in the titre of MSRO strain [52]. Nevertheless, the male-killing NSRO *Spiroplasma* strain was still able to proliferate during aging of adult flies indicating that this strain can resist host immune responses. In contrast, a closely related non male-killing NSRO-A strain could not withstand the host defenses and did also not exhibit immune suppression [56]. 

### 2.2. Modulation of the Host Immune Response Towards Symbionts

Fine-tuning of the host immune response towards endosymbiotic bacteria can also facilitate their maintenance. In *Drosophila* the local immune response of the gut seems to be accurately adjusted for the maintenance of a commensal gut microbiota. The direct contact between gut epithelia and ingested or resident gut bacteria constantly triggers the Imd signaling pathway in the gut. This did not result in AMP production in the gut though, since the intestinal homeobox gene *caudal* represses nuclear factor Kappa B-dependent AMP genes [57]. Furthermore the PGRP-LB plays an important role in bacterial tolerance at epithelial surfaces of *Drosophila* [29]. This catalytic protein is an amidase that specifically cleaves peptidoglycan of Gram-negative bacteria and thereby removes microbial immune elicitors. This negative immune regulation presumably allows modulation of the immune response towards commensal gut bacteria.

An additional possibility to avoid immune actions against symbionts is their compartmentalization into specialized host cells. These so-called bacteriocytes may have an altered gene expression allowing symbionts to persist without triggering an immune response. In the maize weevil *Sitophilus zeamais* the bacteriocytes form an organ, the bacteriome, which harbors the obligate primary endosymbiont (*Sitophilus* primary endosymbiont or SPE) [58]. Suppression subtractive hybridization approaches were used to identify genes differentially expressed within the bacteriome tissue as well as in response to septic injury [59,60]. Interestingly, two putative negative regulators of the immune response were found to be constitutively overexpressed within the bacteriome. One was a gene with sequence homology to *tollip*, a known inhibitor of Toll-like receptor-mediated immune activation in mammals [61]. The other gene, *wpgrp-1*, is a weevil homolog of *Drosophila pgrp-lb* [59]. As aforementioned, this amidase PGRP is known to downregulate the immune response towards commensal gut bacteria [29]. The bacteriome of weevils derives from the gut tissue. Thus, the mechanisms allowing the maintenance of a permanent commensal microbiota in the gut may have evolved to mediate symbiosis in the bacteriome [60]. A recent transcriptome study on *S. oryzae*, the sibling species of *S. zeamais*, also demonstrated that the gene expression profile of bacteriocytes is precisely adjusted to allow tolerance of SPE [62]. In the bacteriome tissue genes involved in cell growth and survival, like the *apoptosis inhibitor *genes *iap2* and *iap3*, the *Rat Sarcoma* (*Ras*) and *leonardo 14-3-3*, were highly expressed. This suggested that weevil bacteriocytes inhibit the apoptosis pathway in order to survive chronic infection with endosymbionts. Furthermore, the expression of immune-relevant genes possibly harming SPE, like *sarcotoxin*, *wpgrp-2*, *gnbp-1* and *c-type lysozyme*, was significantly lower in the bacteriome in comparison to that in larvae without symbionts (aposymbiotic larvae) or to that in larvae challenged with *E. coli*. A striking result of this study was that aposymbiotic insects seem to exhibit a stronger immune response towards pathogens than symbiotic insects, as the genes *sarcotoxin*, *wpgrp-2* and *-3*, *coleoptericin B* and *diptericin* were all induced more strongly upon immune challenge in aposymbiotic than in symbiotic larvae [62]. A possible explanation for the impaired immune competence of symbiotic animals is that endosymbiosis may have selected for a simplification of the host immune system, as already discussed for aphids [62,63]. wPGRP-1 (the weevil homolog of PGRP-LB) apparently plays a key role in preventing activation of the weevil immune system against SPE, as it was not only overexpressed in the *bacteriome* tissue, but also strongly induced in the symbiotic nymphal phase of *S. zeamais* [64]. During this stage the larval bacteriome dissociates and the symbionts are released into the haemolymph [65]. The symbionts must then reach and infect the new adult bacteriome, which is associated with the hindgut. Thus, upregulation of the amidase wPGRP-1 at this stage is likely to prevent immune defenses against the extracellular symbionts through removal of bacterial elicitors [64].

A study on the tsetse fly *G. morsitans morsitans* also demonstrated the importance of PGRP-LB for symbiotic tolerance [66]. In addition to the secondary symbiont *Sodalis*, tsetse flies host the primary obligate endosymbiont *Wigglesworthia glossinidia* within differentiated midgut cells that form a bacteriome [67]. The *pgrp-lb* gene was strongly expressed in this organ and its expression level correlated with symbiont numbers [66]. RNA interference (RNAi)-mediated knock down of *pgrp-lb* expression led to activation of the Imd signaling pathway and resulted in AMP production. The antibacterial activity of the host AMPs finally decreased *Wigglesworthia* number. Thus, PGRP-LB likely scavenges peptidoglycan released by *Wigglesworthia* under normal conditions thereby preventing elimination of the symbiont through the host immune system. Accordingly, it may also contribute to symbiont density regulation. PGRP-LB furthermore seems to play a role in parasite transmission, since reduction in *pgrp-lb* expression increased susceptibility of flies to trypanosome infection [66,68]. Taken together, the data suggest a dual role for PGRP-LB in tsetse flies by mediating the immune response towards symbionts on the one hand and enhancing parasite resistance on the other hand.

An extreme example for symbiont tolerance is the pea aphid *Acyrthosiphon pisum*, which has a reduced immune gene repertoire encoded in its genome in comparison to the known genomes of other insects possibly allowing presence of several different symbiont species [63]. *Buchnera aphidicola* is the primary symbiont of *A. pisum*. This obligate intracellular Gram-negative bacterium provides essential amino acids to its host and is located within bacteriocytes forming a bilobed bacteriome in the aphid haemocoel [1,13,69]. Besides this primary endosymbiont, aphids often harbor one or more secondary facultative endosymbionts extracellularly in the haemolymph, especially the Gram-negative bacteria *Hamiltonella defensa*, *Serratia symbiotica* and *Regiella insecticola* [7,70]. The *A. pisum* genome sequence revealed, that aphids lack most of the genes of the Imd pathway involved in recognition of bacteria (e.g., PGRPs), signal transduction (e.g., Imd or Relish) and antimicrobial response (e.g., AMPs like defensin) [63,71]. Several other studies confirmed that aphids do not raise a strong humoral immune response against bacterial intruders in comparison to other insects [63,72,73,74]. It was discussed that the stably established symbiosis with *Buchnera* might have selected for the loss of immune genes involved in combating Gram-negative bacteria and, as a consequence, also allows the presence of a suite of secondary symbionts [63,75]. However, other insects also harbour several symbionts and still have a complex immune gene repertoire. In fact, a recent study suggested that more sophisticated mechanisms than a mere gene loss are necessary for the maintenance of symbionts within their aphid hosts [74]. It was shown that the aphid cellular immune system is able to eradicate at least some microbial intruders, while leaving symbionts unharmed. This finding implied that aphid symbionts are adapted in a way that they are not recognized or harmed by aphid haemocytes and/or that the aphid immune system is able to distinguish between symbionts and potential pathogens [74].

### 2.3. Control of Bacterial Symbionts

Although endosymbionts are beneficial for their insect hosts and need to be maintained, hosts have to control symbiont number within their tissues in order to prevent their uncontrolled proliferation. Compartmentalization not only allows maintenance of symbionts unharmed from immune attacks, but also helps to restrict their occurrence to a specialized host tissue. It was shown that intracellular symbionts like SPE from weevils [60] or *Blochmannia floridanus* from carpenter ants [76] are still recognized and possibly attacked by the host immune system, when injected into the haemocoel. Thus, the immune system is probably also involved in controlling the symbiont population and prevents symbionts from escaping bacteriocytes. Accordingly, weevils have to downregulate the immune response towards SPE during its extracellular phase [64]. The prerequisite for symbiont detection and regulation presumably is the presence of MAMPs on the bacterial surface, as SPE and *Blochmannia* still have a (reduced) cell wall in contrast to wall-free *Spiroplasma* [34,60,76]. 

In the past, it was often argued that intracellular symbionts would be shielded from the immune system, because recognition and effector proteins of known innate immune signaling pathways, like Toll or Imd, are secreted into the haemolymph and thus only act on extracellular bacteria. However, it is also known that autophagy can act as an intracellular innate immune mechanism protecting against intracellular bacteria [77]. Autophagy is a cellular pathway for protein and organelle turnover involving degradation of cell components through the lysosomal system. The material for degradation is first separated from the rest of the cytoplasm by double- or multiple membrane structures. These so-called autophagosomes then fuse with lysosomes, which provide hydrolases for degradation [77,78,79]. In *Drosophila* it was shown that autophagy also acts as an innate defense mechanism against intracellular pathogens, such as *Listeria monocytogenes*, and is induced independently from Toll and Imd signaling pathways [80]. The PRR PGRP-LE mediates the detection of intracellular bacteria with DAP-type peptidoglycan, which induces autophagy and independently from that also activates the Imd pathway resulting in AMP production [80]. Thus, autophagy and degradation via the lysosomal system might also be involved in controlling intracellular symbionts. Accordingly weevil bacteriocytes highly express several genes involved in vesicular formation and trafficking possibly supporting autophagocytic processes [59,62]. A transcriptome study in aphids revealed that bacteriocytes, harboring *Buchnera* symbionts, overexpress a *lysozyme* gene [81]. This finding is consistent with former electron microscopy images showing that *Buchnera* are occasionally degraded by lysosomes [69,82]. Furthermore, recent studies demonstrated a connection between *Buchnera* degradation and age- and morph-dependent activation of the lysosomal machinery [83]. In aphids the number of bacteriocytes and of *Buchnera* cells within them varied in relation to age and polyphenism of the aphid host [84,85]. In winged aphids the *Buchnera* density decreased drastically around the final ecdysis [83]. As this decrease in symbiont number was much stronger in winged than in unwinged aphids and occurred simultaneously to the rapid development of flight muscles, the degradation of *Buchnera* might provide larger amounts of nutrients to the host needed for the formation of flight muscles [83,84]. In winged aphids the number of lysosome-like acidic organelles increased drastically in the bacteriocytes and also the expression of lysosome-related genes (encoding lysozyme and cathepsin L) was strongly induced, suggesting *Buchnera* degradation proceeds through the lysosomal system [83]. An addition, a host serine carboxypeptidase was identified that seems to be involved in proteolytic activation of *Buchnera*-degrading enzymes and/or directly in digesting *Buchnera* proteins in the lysosomes of bacteriocytes in alatae [86]. To satisfy host demands during this important developmental stage, degradation of *Buchnera* most likely enhances host fitness and thereby indirectly also the fitness of *Buchnera* symbionts [86]. It is known that the endosymbiont populations of other insects also alternate in relation to developmental stage and/or physiological condition. For example, in the carpenter ant *Camponotus floridanus* the distribution of bacteriocytes and of *Blochmannia floridanus* endosymbionts varied strongly over different developmental stages [87]. The percentage of endosymbiont-bearing midgut cells increased strongly during host ontogeny and peaks in late pupal stages, where the entire midgut is transformed into a symbiotic organ. Accordingly, symbionts seem to have an important function in this developmental phase during which ant hosts are enclosed from the environment via the puparium and no external food is ingested [87,88]. After eclosion of workers the bacterial number decreased with increasing age of workers. Similar to the aphid-*Buchnera*-symbiosis autophagy might be a mechanism for controlling intracellular *Blochmannia* [83,87] and to supply the ant host with essential nutrients, as *Blochmannia* has been shown to contribute to host nutrition by production of essential amino acids [89].

A recent study demonstrated the possibility to control intracellular endosymbionts via host AMPs [90]. Gene expression studies revealed overexpression of the AMP gene *coleoptericin-A* (*colA*) in bacteriocytes of *Sitophilus* weevils which host the intracellular Gram-negative γ-Proteobacterium SPE [60,62,91]. Using immunohistochemistry with an antibody against ColA, Login *et al*. (2011) confirmed that *colA* is expressed in all endosymbiont-bearing tissues. The ColA peptide was even located inside the SPE cytoplasm and sometimes also found to be attached to the bacterial membrane surface. In antimicrobial activity assays ColA showed bactericidal activity against Gram-positive *Micrococcus luteus* and Gram-negative *E. coli*. Interestingly, at bacteriostatic conditions ColA inhibited cell division and caused bacterial gigantism only in *E. coli*, but not in *M. luteus*. The resulting giant cell phenotype resembles the elongated morphology of SPE. The authors then used far-Western blotting in order to identify bacterial molecules targeted by ColA peptides [90]. Amongst others, ColA was found to interact with bacterial Omps and with GroEL. Presumably, ColA enters the bacterial cytoplasm via bacterial Omps and then causes cell elongation through interaction with GroEL, because *groEL* mutations in *E. coli* are also known to elicit cell gigantism [92]. When *colA* transcripts in larval weevils were knocked down by RNAi, cytokinesis was resumed and the elongated form of SPE lost again [90]. As a consequence SPE was able to escape from bacteriocytes and spread through larval tissues. Taken together, the weevil ColA peptide has a special symbiotic function in weevil endosymbiosis by controlling endosymbiont number and location. With respect to its bacteriostatic and bactericidal activities against *E. coli* and *M. luteus*, ColA likely also accomplishes a dual function in weevils, as already discussed for PGRP-LB in tsetse flies.

## 3. Positive Effects of Symbionts on Host Immune Competence

### 3.1. Protective Symbiosis

Downregulation of the host immune response in order to maintain endosymbionts might have negative effects on host immune competence against pathogens. However, endosymbionts have been reported to confer resistance towards pathogens and parasites in several insects (reviewed in [93,94]). Prominent examples for such symbiont-mediated resistance have been described in aphids. Secondary endosymbionts, especially *H. defensa* and partially also *S. symbiotica* and *R. insecticola*, have been shown to protect several aphid host species against attacks from the parasitoids *Aphidius ervi* and *A. eadyi* [7,9,95,96,97,98]. This protective effect of facultative symbionts was first described in *A. pisum*, which is often parasitized by *A. ervi *[95]. Female wasps lay their eggs into the body cavity of aphid nymphs, where the parasitoid larvae then develop and finally often kill their aphid host [99]. Infection of *A. pisum* with *S. symbiotica* and in particular with *H. defensa* enhanced resistance to parasitoid attack by increasing mortality of developing parasitoid larvae [9,95]. Furthermore, the fecundity of parasitized aphids carrying *H. defensa* (but not *S. symbiotica*) was significantly increased in comparison to parasitized uninfected animals [100]. This direct fitness benefit of *H. defensa* infection was also reflected in the rapid increase of infection frequency in experimental populations when the parasitoid wasp was present [101]. In the absence of parasitism the frequencies of *Hamiltonella*-infected aphids declined again, indicating a probable cost to infection [101]. Depending on the *H. defensa* strain the protection levels vary from small reduction in successful parasitism to complete resistance. Recent studies indicated that the protective effect of *H. defensa* depends on the presence and on the variant of a bacteriophage called APSE (*A. pisum* secondary endosymbiont bacteriophage), which infects the symbiont [7,8,102,103]. APSE variants encode different putative toxins with homology to known toxins from vertebrate pathogens. Amongst them are a cytolethal distending toxin, a Shiga-like toxin and a YD-repeat toxin, which possibly target eukaryotic tissue, in this case the presumably developing parasitoid wasp [102,103]. Besides the protection against wasps, *R. insecticola*, another secondary endosymbiont of aphids, has also been shown to enhance host resistance to the aphid-specific fungal entomopathogen *Pandora neoaphidis* [98,104]. Thus, all three common secondary endosymbionts of *A. pisum* (*H. defensa*, *S. symbiotica*, *R. insecticola*) can confer defense traits towards their hosts. These protective symbiotic interactions of aphids may compensate for the limited humoral immune response resulting from the reduction of immune gene repertoire [63].

Symbiont-mediated protection has also been described in several other insect-symbiont systems [94]. In *Drosophila hydei* for example, resistance against parasitic wasps is mediated via *Spiroplasma* [105]. Furthermore, these symbionts protect *Drosophila neotestacea* against sterilization through a parasitic nematode [106] and may thus rapidly spread through a host population [107]. Moreover, endosymbionts could aid their hosts against fungal and bacterial pathogens through the production of antibiotics, which has already been shown for digger wasps [6,108] as well as for fungus-growing attine ants [109] and pine beetles [110]. Aside from the protection against pathogens and parasites endosymbionts may also protect their hosts from predators. For example endosymbionts of *Paederus* beetles produce the polyketide pederin, a toxin that protects *Paederus* larvae from wolf spiders by reducing their palatability as prey [111,112,113].

As aforementioned, *Wolbachia* bacteria are remarkably widespread reproductive parasites of arthropods [35,38]. A study on *D. melanogaster*, however, suggested that under field conditions *Wolbachia*-transmission due to reproductive manipulation alone is not entirely sufficient to invade host populations. Therefore it was predicted that *Wolbachia* must additionally confer a fitness benefit to its host in order to ensure its persistence [114]. Recently, it has been shown that *Wolbachia* infection renders *Drosophila* more resistant against diverse RNA viruses such as *Drosophila* C virus, Flock House Virus and Nora Virus [10,115]. This antiviral effect of *Wolbachia* infection is also effective in other dipterans like *Culex quinquefasciatus* or *Aedes aegypti* and might therefore be applied to reduce their competence as vector for viral diseases like West Nile, Dengue or Chikungunya [40,116]. Aside from virus protection the *Wolbachia* strain wMelPop furthermore inhibits *Plasmodium* development in important *Anopheles* vector species and also has an inhibitory effect on filarial nematodes in *Aedes* mosquitos [41,117]. Thus *Wolbachia*-mediated protection might become a new tool to develop novel control strategies for vector-borne diseases like malaria or filariasis.

### 3.2. Immune Priming via Symbionts

The inhibitory effects of *Wolbachia* infection against various pathogens seem to result from priming of the host immune system by these bacteria. Host gene expression analyses revealed upregulation of several immune genes in response to *Wolbachia* infection. Amongst others these included genes that encode thioester-containing proteins (TEPs), C-type lectins fibrinogen-related proteins (FREPs), cecropins, defensins and a leucine-rich repeat immune protein (LRIM1) [40,41,117]. The induction of these genes seems to be a possible explanation for the observed antiviral effects, as for example cecropins have already been shown to inhibit viral replication [118,119]. Cecropins furthermore inhibited *Plasmodium* development in *Anopheles gambiae* [120,121] and a protective effect against *Plasmodium*-infection has also been shown for genes *tep1* and *lrim1* by dsRNA-mediated gene knockdown [41,122]. Actually for the *tep1* gene a direct causal connection between *Wolbachia*-mediated gene upregulation and *Plasmodium* inhibition has been demonstrated. Disruption of *tep1* gene expression by simultaneous injection of *Wolbachia* and *tep1*-dsRNA resulted in significantly higher oocyst numbers in *tep1*-knockdown animals in comparison to control animals, which were injected with *Wolbachia* at the same time as with unspecific dsRNA [41]. Thus, in certain host species *Wolbachia* infection seems to trigger expression of some host immune genes that prevent colonization of host tissues by other pathogens.

There is emerging evidence that symbionts play an important role in shaping diverse immune functions. Recent studies on *Glossina morsitans* showed that tsetse flies require the presence of endosymbionts (especially *Wigglesworthia*) during larval development for maturation and subsequent proper functioning of their immune system [123,124]. *G. morsitans* hosts harbour several different bacterial symbionts. These include the secondary symbionts *Sodalis* and *Wolbachia* as well as the obligate mutualist *Wigglesworthia*, which are maternally transmitted to the host offspring throughout milk gland secretions during the intrauterine development [125,126]. In adult flies the primary symbiont *Wigglesworthia* resides in a bacteriome at the anterior gut and provides its host with certain vitamins lacking from the vertebrate blood diet [2,127]. *Glossina* larvae, lacking *Wigglesworthia* (*Gmm^Wgm-^*) but still harboring *Sodalis* and *Wolbachia*, were obtained by feeding pregnant female tsetse flies with the antibiotic ampicillin [68,123]. Tetracycline treatment resulted in aposymbiotic tsetse (*Gmm^Apo^*) lacking all endosymbionts [68,124]. Interestingly, adult *Gmm^Wgm-^* and especially *Gmm^Apo^* flies were highly susceptible to *E. coli* infection in comparison to wild-type flies (*Gmm^WT^*) harboring all endosymbionts [123,124]. This effect was observed only when the antibiotic treatment was conducted during the larval stage, as flies whose microbiotia was eliminated only during the adult stage (*Gmm^WT/Wgm-^*) were almost as resistant to *E. coli* infection as to the wild-type flies (*Gmm^WT^*). This result implied that *Wigglesworthia* is not directly responsible for resistance against *E. coli*, but rather has to be present during larval stages in order to stimulate immune system development in adults [123]. Accordingly, immune system development could be partially achieved in aposymbiotic offspring, when their symbiont-free mothers were fed with a diet supplemented with *Wigglesworthia* cell extracts (*Gmm^Apo/Wgm^*) [124]. Feeding of *Sodalis* cell extracts (*Gmm^Apo/Sgm^*) did not yield the same effect suggesting that specific molecular components of the obligate endosymbiont *Wigglesworthia* exhibit immunostimulatory activity within tsetse hosts [124]. Presence of symbionts triggers expression of several immune genes during larval development, as the negative immune regulator *caudal*, the JAK/STAT pathway receptor *domeless* and the *dual oxidase* (*DUOX*) gene were significantly less expressed in uninfected mature *Gmm^WT^* than in *Gmm^Apo^* flies [124]. After infection with *E. coli*, immune gene expression in mature *Gmm^WT^* differed strongly from that in *Gmm^Apo^* and *Gmm^Wgm-^* flies. The expression of genes associated with humoral immune responses, like the AMP genes *cecropin* and *attacin*, was less induced in *Gmm^Wgm-^* than in *Gmm^WT^*, while in contrast the same genes were significantly upregulated in *Gmm^Apo^* compared with *Gmm^WT^*. Genes associated with cellular immune processes like phagocytosis and melanization were significantly downregulated in both *Gmm^Apo^* and *Gmm^Wgm-^* compared with *Gmm^WT^* [123,124]. Particularly, *prophenoloxidase* (*PPO*) gene was not induced in *Gmm^Apo^* and *Gmm^Wgm-^* after inoculation with *E. coli* and neither hemolymph clotting nor melanin deposition could be seen at the wound side. *Gmm^Apo^* and *Gmm^Wgm-^* flies seemed to have a significantly reduced number of haemocytes with homologous function to *Drosophila* crystal cells [123,124], which produce and store PPO and release this protein upon injury in order to facilitate melanin deposition [17,128]. The failure of *Gmm^Apo^* and *Gmm^Wgm-^* to induce expression of two *thioester-containing protein* genes (*tep2* and *tep4*) upon *E. coli*-infection suggested a compromised ability for phagocytosis [123,124], as TEPs likely opsonize bacterial cells and thus promote phagocytosis in insects [129]. The authors demonstrated the importance of phagocytosis as defense mechanism against *E. coli* and showed that *Gmm^WT^* have over three times more circulating haemocytes/µL of haemolymph than mature *Gmm^Wgm-^* and over hundred times more than mature *Gmm^Apo^* flies [123,124]. Furthermore, the high susceptibility of *Gmm^Apo^* flies against normally nonpathogenic *E. coli* could be reversed by transplantation of haemocytes from *Gmm^WT^* to *Gmm^Apo^* flies providing an explicit correlation between the fly´s haemocytes and its immune competence [124]. In accordance with that two conserved transcription factors, *lozenge* and *serpent*, known to be essential for hematopoiesis in *Drosophila *[130], were significantly less expressed in *Gmm^Apo^* and *Gmm^Wgm-^* than in *Gmm^WT^* larvae [123,124]. Expression of these transcription factors and thereby hematopoiesis could be stimulated in intrauterine aposymbiotic larvae by feeding their mothers with cell extracts from *Wigglesworthia* (*Gmm^Apo/Wgm^*), but not from *Sodalis* (*Gmm^Apo/Sgm^*) [124]. Taken together, these studies demonstrated that presence of endosymbionts, especially of obligate *Wigglesworthia*, in larvae is needed for complete development and function of cellular immune system in adult tsetse flies [123,124]. The astonishing function of *Wigglesworthia* may have evolved based on the relatively aseptic life style of tsetse flies due to their sterile vertebrate blood diet and viviparous reproduction [123]. Besides, other studies also confirmed a positive effect of *Wigglesworthia* on tsetse immune competence in terms of reducing susceptibility to trypanosome infection probably by stimulating *pgrp-lb* expression [66,68].

## 4. Conclusion and Future Directions

So far we still do not have a comprehensive view of the mechanisms leading to establishment and maintenance of bacterial endosymbiosis in insects. Comparing the data from different model systems highlights the fact that there are several possible adaptations on the symbiont side as well as on the host side leading to symbiont tolerance. On the one hand symbionts may have reduced or altered immune elicitors and/or may be able to modulate the host immune system e.g., via secretion of effector proteins. On the other hand the host may reduce defense actions against symbionts via their compartmentalization into specialized host tissues with relaxed immune sanctions and adapted symbiont control mechanisms. By doing so, the host also prevents uncontrolled spreading of symbionts through its tissues. Several studies on different insects indicate a key role for amidase PGRPs, especially PGRP-LB, in downregulation of the immune response towards symbionts [29,64,66] and the involvement of autophagocytic processes in symbiont control [42,62,83].

Recently published transcriptomic studies revealed that chronic infection with endosymbionts affects several different cellular functions, such as oxidative stress regulation, immune pathways, apoptosis and autophagy [42,62]. As all these function are also known to be affected in host-pathogen interactions, it was suggested that a common language exists between bacteria and their hosts. The cellular pathways may be affected differently as a function of the mode of the bacteria-host-interaction as well as according to the bacterial location within the host [42]. More detailed and comparative analyses between host-pathogen- and host-symbiont-interactions as well as between ancient and more recent symbioses are needed to elucidate the molecular mechanisms enabling the establishment of symbiosis. In symbiotic relationships with a long co-evolutionary history host defense molecules seem to have been selected for a special symbiotic function, as seen for ColA in weevils [90] and PGRP-LB in tsetse flies [66]. Next generation sequencing in combination with RNAi technology provide powerful tools to identify and characterize new symbiosis-relevant genes, even in species, where no reference genome is available. For example in weevils several gene sequences with unknown function but high expression in symbiont-full bacteriocytes have already been identified and need to be further characterized [62]. Comparison of such gene sets with data from many other insects with different evolutionary background may help to identify conserved hypothetical genes with relevance for symbiosis. 

Future studies also need to focus on the role of the cellular immune response in mediating host-symbiont interactions, as several studies indicate the importance of this more sophisticated part of the innate defense system [74,123,124,131]. 

Another interesting aspect that needs to be investigated more intensively is the impact of symbiosis on host immune competence in general, because so far the data indicate symbiosis specific mechanisms. In weevils symbiotic larvae exhibit a compromised immune response in comparison to aposymbiotic larvae, while in contrast tsetse larvae require the presence of endosymbionts for maturation of their immune system [62,123,124]. Several studies report symbiont-mediated protection against various pathogens and thus symbionts may even be considered part of the host immune system [93,94,132]. The few insect-symbiont systems investigated so far indicate highly specific mechanisms leading to symbiont tolerance in dependence of symbiont tissue location and of the proportion of intra- to extracellular phases. Thus, there is urgent need to study several different systems to uncover the variety of molecular tolerance mechanisms as well as to infer general principles. In sum, it is obvious that the presence of symbionts definitely affects the host immune system. Moreover, symbionts likely played an important role in the evolution of the insect immune system. A better understanding of the role of endosymbionts in shaping host immune functions will contribute to the development of novel symbiont-based strategies for the control of insect-borne diseases and can lead to new insights how microorganisms interact with the innate immune system.

## References

[1] Douglas A.E. (1998). Nutritional interactions in insect-microbial symbioses: Aphids and their symbiotic bacteria Buchnera. Annu. Rev. Entomol..

[2] Akman L., Yamashita A., Watanabe H., Oshima K., Shiba T., Hattori M., Aksoy S. (2002). Genome sequence of the endocellular obligate symbiont of tsetse flies, *Wigglesworthia glossinidia*. Nat. Genet..

[3] Zientz E., Dandekar T., Gross R. (2004). Metabolic interdependence of obligate intracellular bacteria and their insect hosts. Microbiol. Mol. Biol. Rev..

[4] Dunbar H.E., Wilson A.C., Ferguson N.R., Moran N.A. (2007). Aphid thermal tolerance is governed by a point mutation in bacterial symbionts. PLoS Biol..

[5] Kaltenpoth M. (2009). Actinobacteria as mutualists: General healthcare for insects?. Trends Microbiol..

[6] Kaltenpoth M., Gottler W., Herzner G., Strohm E. (2005). Symbiotic bacteria protect wasp larvae from fungal infestation. Curr. Biol..

[7] Oliver K.M., Degnan P.H., Burke G.R., Moran N.A. (2010). Facultative symbionts in aphids and the horizontal transfer of ecologically important traits. Annu. Rev. Entomol..

[8] Oliver K.M., Degnan P.H., Hunter M.S., Moran N.A. (2009). Bacteriophages encode factors required for protection in a symbiotic mutualism. Science.

[9] Oliver K.M., Moran N.A., Hunter M.S. (2005). Variation in resistance to parasitism in aphids is due to symbionts not host genotype. Proc. Natl. Acad. Sci. USA.

[10] Teixeira L., Ferreira A., Ashburner M. (2008). The bacterial symbiont *Wolbachia* induces resistance to RNA viral infections in *Drosophila melanogaster*. PLoS Biol..

[11] Brownlie J.C., Johnson K.N. (2009). Symbiont-mediated protection in insect hosts. Trends Microbiol..

[12] Currie C.R., Wong B., Stuart A.E., Schultz T.R., Rehner S.A., Mueller U.G., Sung G.H., Spatafora J.W., Straus N.A. (2003). Ancient tripartite coevolution in the attine ant-microbe symbiosis. Science.

[13] Baumann P. (2005). Biology bacteriocyte-associated endosymbionts of plant sap-sucking insects. Annu. Rev. Microbiol..

[14] Braendle C., Miura T., Bickel R., Shingleton A.W., Kambhampati S., Stern D.L. (2003). Developmental origin and evolution of bacteriocytes in the aphid-*Buchnera* symbiosis. PLoS Biol..

[15] Dale C., Moran N.A. (2006). Molecular interactions between bacterial symbionts and their hosts. Cell.

[16] Russell J.A., Latorre A., Sabater-Munoz B., Moya A., Moran N.A. (2003). Side-stepping secondary symbionts: Widespread horizontal transfer across and beyond the Aphidoidea. Mol. Ecol..

[17] Lemaitre B., Hoffmann J. (2007). The host defense of *Drosophila melanogaster*. Annu. Rev. Immunol..

[18] Feldhaar H., Gross R. (2008). Immune reactions of insects on bacterial pathogens and mutualists. Microbes Infect..

[19] Dziarski R. (2004). Peptidoglycan recognition proteins (PGRPs). Mol. Immunol..

[20] Royet J., Dziarski R. (2007). Peptidoglycan recognition proteins: Pleiotropic sensors and effectors of antimicrobial defences. Nat. Rev. Microbiol..

[21] Gottar M., Gobert V., Matskevich A.A., Reichhart J.M., Wang C., Butt T.M., Belvin M., Hoffmann J.A., Ferrandon D. (2006). Dual detection of fungal infections in *Drosophila* via recognition of glucans and sensing of virulence factors. Cell.

[22] Lee W.J., Lee J.D., Kravchenko V.V., Ulevitch R.J., Brey P.T. (1996). Purification and molecular cloning of an inducible gram-negative bacteria-binding protein from the silkworm, *Bombyx mori*. Proc. Natl. Acad. Sci. USA.

[23] Kim Y.S., Ryu J.H., Han S.J., Choi K.H., Nam K.B., Jang I.H., Lemaitre B., Brey P.T., Lee W.J. (2000). Gram-negative bacteria-binding protein, a pattern recognition receptor for lipopolysaccharide and beta-1,3-glucan that mediates the signaling for the induction of innate immune genes in *Drosophila melanogaster* cells. J. Biol. Chem..

[24] Delaney J.R., Stoven S., Uvell H., Anderson K.V., Engstrom Y., Mlodzik M. (2006). Cooperative control of *Drosophila* immune responses by the JNK and NF-kappaB signaling pathways. EMBO J..

[25] Boutros M., Agaisse H., Perrimon N. (2002). Sequential activation of signaling pathways during innate immune responses in *Drosophila*. Dev. Cell..

[26] Moran N.A., McCutcheon J.P., Nakabachi A. (2008). Genomics and evolution of heritable bacterial symbionts. Annu. Rev. Genet..

[27] Rio R.V., Wu Y.N., Filardo G., Aksoy S. (2006). Dynamics of multiple symbiont density regulation during host development: Tsetse fly and its microbial flora. Proc. R. Soc. Lond B Biol. Sci..

[28] Weiss B.L., Wu Y., Schwank J.J., Tolwinski N.S., Aksoy S. (2008). An insect symbiosis is influenced by bacterium-specific polymorphisms in outer-membrane protein A. Proc. Natl. Acad. Sci. USA.

[29] Zaidman-Remy A., Herve M., Poidevin M., Pili-Floury S., Kim M.S., Blanot D., Oh B.H., Ueda R., Mengin-Lecreulx D., Lemaitre B. (2006). The *Drosophila* amidase PGRP-LB modulates the immune response to bacterial infection. Immunity.

[30] Hao Z., Kasumba I., Lehane M.J., Gibson W.C., Kwon J., Aksoy S. (2001). Tsetse immune responses and trypanosome transmission: Implications for the development of tsetse-based strategies to reduce trypanosomiasis. Proc. Natl. Acad. Sci. USA.

[31] Hu Y., Aksoy S. (2005). An antimicrobial peptide with trypanocidal activity characterized from *Glossina morsitans morsitans*. Insect Biochem. Mol. Biol..

[32] Dale C., Young S.A., Haydon D.T., Welburn S.C. (2001). The insect endosymbiont *Sodalis glossinidius* utilizes a type III secretion system for cell invasion. Proc. Natl. Acad. Sci. USA.

[33] Bourtzis K., Pettigrew M.M., O’Neill S.L. (2000). *Wolbachia* neither induces nor suppresses transcripts encoding antimicrobial peptides. Insect Mol. Biol..

[34] Hurst G.D., Anbutsu H., Kutsukake M., Fukatsu T. (2003). Hidden from the host: *Spiroplasma* bacteria infecting *Drosophila* do not cause an immune response, but are suppressed by ectopic immune activation. Insect Mol. Biol..

[35] Hilgenboecker K., Hammerstein P., Schlattmann P., Telschow A., Werren J.H. (2008). How many species are infected with *Wolbachia*?-A statistical analysis of current data. FEMS Microbiol. Lett..

[36] Stouthamer R., Breeuwer J.A., Hurst G.D. (1999). *Wolbachia pipientis*: Microbial manipulator of arthropod reproduction. Annu. Rev. Microbiol..

[37] Werren J.H. (1997). Biology of *Wolbachia*. Annu. Rev. Entomol..

[38] Werren J.H., Baldo L., Clark M.E. (2008). *Wolbachia*: Master manipulators of invertebrate biology. Nat. Rev. Microbiol..

[39] Xi Z., Gavotte L., Xie Y., Dobson S.L. (2008). Genome-wide analysis of the interaction between the endosymbiotic bacterium *Wolbachia* and its *Drosophila* host. BMC Genomics.

[40] Moreira L.A., Iturbe-Ormaetxe I., Jeffery J.A., Lu G., Pyke A.T., Hedges L.M., Rocha B.C., Hall-Mendelin S., Day A., Riegler M. et al. (2009). A *Wolbachia* symbiont in *Aedes aegypti* limits infection with dengue, *Chikungunya*, and *Plasmodium*. Cell.

[41] Kambris Z., Blagborough A.M., Pinto S.B., Blagrove M.S., Godfray H.C., Sinden R.E., Sinkins S.P. (2010). *Wolbachia* stimulates immune gene expression and inhibits *Plasmodium* development in *Anopheles gambiae*. PLoS Pathog..

[42] Kremer N., Charif D., Henri H., Gavory F., Wincker P., Mavingui P., Vavre F. (2012). Influence of *Wolbachia* on host gene expression in an obligatory symbiosis. BMC Microbiol..

[43] Wu M., Sun L.V., Vamathevan J., Riegler M., Deboy R., Brownlie J.C., McGraw E.A., Martin W., Esser C., Ahmadinejad N. et al. (2004). Phylogenomics of the reproductive parasite *Wolbachia pipientis* wMel: A streamlined genome overrun by mobile genetic elements. PLoS Biol..

[44] Sedgwick S.G., Smerdon S.J. (1999). The ankyrin repeat: A diversity of interactions on a common structural framework. Trends Biochem. Sci..

[45] McGraw E.A., O’Neill S.L. (2004). *Wolbachia pipientis*: Intracellular infection and pathogenesis in *Drosophila*. Curr. Opin. Microbiol..

[46] Pan X., Luhrmann A., Satoh A., Laskowski-Arce M.A., Roy C.R. (2008). Ankyrin repeat proteins comprise a diverse family of bacterial type IV effectors. Science.

[47] Thoetkiattikul H., Beck M.H., Strand M.R. (2005). Inhibitor kappaB-like proteins from a polydnavirus inhibit NF-kappaB activation and suppress the insect immune response. Proc. Natl. Acad. Sci. USA.

[48] Regassa L.B., Gasparich G.E. (2006). *Spiroplasmas*: Evolutionary relationships and biodiversity. Front Biosci..

[49] Sakaguchi B., Poulson D.F. (1961). Distribution of “sex-ratio” agent in tissues of *Drosophila willistoni*. Genetics.

[50] Montenegro H., Solferini V.N., Klaczko L.B., Hurst G.D. (2005). Male-killing *Spiroplasma* naturally infecting *Drosophila melanogaster*. Insect Mol. Biol..

[51] Counce S.J., Poulson D.F. (1961). The Developmental Effects of Hereditary Infections in *Drosophila*. Am. Zool..

[52] Herren J.K., Lemaitre B. (2011). *Spiroplasma* and host immunity: Activation of humoral immune responses increases endosymbiont load and susceptibility to certain Gram-negative bacterial pathogens in *Drosophila melanogaster*. Cell Microbiol..

[53] Hutchence K.J., Fischer B., Paterson S., Hurst G.D. (2011). How do insects react to novel inherited symbionts? A microarray analysis of *Drosophila melanogaster* response to the presence of natural and introduced *Spiroplasma*. Mol. Ecol..

[54] Whitcomb R.F., Williamson D.L. (1975). Helical wall-free prokaryotes in insects: Multiplication and pathogenicity. Ann. N. Y. Acad. Sci..

[55] Aggrawal K., Silverman N. (2007). Peptidoglycan recognition in *Drosophila*. Biochem. Soc. Trans..

[56] Anbutsu H., Fukatsu T. (2010). Evasion, suppression and tolerance of *Drosophila* innate immunity by a male-killing *Spiroplasma* endosymbiont. Insect Mol. Biol..

[57] Ryu J.H., Kim S.H., Lee H.Y., Bai J.Y., Nam Y.D., Bae J.W., Lee D.G., Shin S.C., Ha E.M., Lee W.J. (2008). Innate immune homeostasis by the homeobox gene *caudal* and commensal-gut mutualism in *Drosophila*. Science.

[58] Heddi A., Grenier A.M., Khatchadourian C., Charles H., Nardon P. (1999). Four intracellular genomes direct weevil biology: Nuclear, mitochondrial, principal endosymbiont, and *Wolbachia*. Proc. Natl. Acad. Sci. USA.

[59] Heddi A., Vallier A., Anselme C., Xin H., Rahbe Y., Wackers F. (2005). Molecular and cellular profiles of insect bacteriocytes: Mutualism and harm at the initial evolutionary step of symbiogenesis. Cell Microbiol..

[60] Anselme C., Perez-Brocal V., Vallier A., Vincent-Monegat C., Charif D., Latorre A., Moya A., Heddi A. (2008). Identification of the weevil immune genes and their expression in the bacteriome tissue. BMC Biol..

[61] Zhang G., Ghosh S. (2002). Negative regulation of toll-like receptor-mediated signaling by Tollip. J. Biol. Chem..

[62] Vigneron A., Charif D., Vincent-Monegat C., Vallier A., Gavory F., Wincker P., Heddi A. (2012). Host gene response to endosymbiont and pathogen in the cereal weevil *Sitophilus oryzae*. BMC Microbiol..

[63] Gerardo N.M., Altincicek B., Anselme C., Atamian H., Barribeau S.M., de Vos M., Duncan E.J., Evans J.D., Gabaldon T., Ghanim M. et al. (2010). Immunity and other defenses in pea aphids, *Acyrthosiphon pisum*. Genome. Biol..

[64] Anselme C., Vallier A., Balmand S., Fauvarque M.O., Heddi A. (2006). Host PGRP gene expression and bacterial release in endosymbiosis of the weevil *Sitophilus zeamais*. Appl. Environ. Microbiol..

[65] Buchner P. (1965). Endosymbiosis of animals with plant microorganisms.

[66] Wang J., Wu Y., Yang G., Aksoy S. (2009). Interactions between mutualist *Wigglesworthia* and tsetse peptidoglycan recognition protein (PGRP-LB) influence trypanosome transmission. Proc. Natl. Acad. Sci. USA.

[67] Aksoy S. (1995). *Wigglesworthia* gen. nov. and *Wigglesworthia glossinidia* sp. nov., taxa consisting of the mycetocyte-associated, primary endosymbionts of tsetse flies. Int. J. Syst. Bacteriol..

[68] Pais R., Lohs C., Wu Y., Wang J., Aksoy S. (2008). The obligate mutualist *Wigglesworthia* glossinidia influences reproduction, digestion, and immunity processes of its host, the tsetse fly. Appl. Environ. Microbiol..

[69] Griffith G.W., Beck S.D. (1973). Intracellular symbiotes of pea aphid, *Acyrthosiphon pisum*. J. Insect Physiol..

[70] Moran N.A., Russell J.A., Koga R., Fukatsu T. (2005). Evolutionary relationships of three new species of Enterobacteriaceae living as symbionts of aphids and other insects. Appl. Environ. Microbiol..

[71] Consortium I.A.G. (2010). Genome sequence of the pea aphid *Acyrthosiphon pisum*. PLoS Biol..

[72] Altincicek B., Gross J., Vilcinskas A. (2008). Wounding-mediated gene expression and accelerated viviparous reproduction of the pea aphid *Acyrthosiphon pisum*. Insect Mol. Biol..

[73] Burke G.R., Moran N.A. (2011). Responses of the pea aphid transcriptome to infection by facultative symbionts. Insect Mol. Biol..

[74] Laughton A.M., Garcia J.R., Altincicek B., Strand M.R., Gerardo N.M. (2011). Characterisation of immune responses in the pea aphid, *Acyrthosiphon pisum*. J. Insect Physiol..

[75] Douglas A.E., Bouvaine S., Russell R.R. (2011). How the insect immune system interacts with an obligate symbiotic bacterium. Proc. R. Soc. Lond B Biol. Sci..

[76] Ratzka C., Liang C., Dandekar T., Gross R., Feldhaar H. (2011). Immune response of the ant *Camponotus floridanus* against pathogens and its obligate mutualistic endosymbiont. Insect Biochem. Mol. Biol..

[77] Amano A., Nakagawa I., Yoshimori T. (2006). Autophagy in innate immunity against intracellular bacteria. J. Biochem..

[78] Kirkegaard K., Taylor M.P., Jackson W.T. (2004). Cellular autophagy: Surrender, avoidance and subversion by microorganisms. Nat. Rev. Microbiol..

[79] Mizushima N., Levine B., Cuervo A.M., Klionsky D.J. (2008). Autophagy fights disease through cellular self-digestion. Nature.

[80] Yano T., Mita S., Ohmori H., Oshima Y., Fujimoto Y., Ueda R., Takada H., Goldman W.E., Fukase K., Silverman N. et al. (2008). Autophagic control of *Listeria* through intracellular innate immune recognition in *Drosophila*. Nat. Immunol..

[81] Nakabachi A., Shigenobu S., Sakazume N., Shiraki T., Hayashizaki Y., Carninci P., Ishikawa H., Kudo T., Fukatsu T. (2005). Transcriptome analysis of the aphid bacteriocyte, the symbiotic host cell that harbors an endocellular mutualistic bacterium, *Buchnera*. Proc. Natl. Acad. Sci. USA.

[82] Hinde R. (1971). Control of mycetome symbiotes of aphids *Brevicoryne brassicae*, *Myzus persicae*, and *Macrosiphum rosa*. J. Insect Physiol..

[83] Nishikori K., Morioka K., Kubo T., Morioka M. (2009). Age- and morph-dependent activation of the lysosomal system and *Buchnera* degradation in aphid endosymbiosis. J. Insect Physiol..

[84] Hongoh Y., Ishikawa H. (1994). Changes of mycetocyte symbiosis in response to flying behavior of alatiform aphid (*Acyrthosiphon pisum*). Zool. Sci..

[85] Douglas A.E., Dixon A.F.G. (1987). The mycetocyte symbiosis of aphids - Variation with age and morph in virginoparae of *Megoura viciae* and *Acyrthosiphon pisum*. J. Insect Physiol..

[86] Nishikori K., Kubo T., Morioka M. (2009). Morph-dependent expression and subcellular localization of host serine carboxypeptidase in bacteriocytes of the pea aphid associated with degradation of the endosymbiotic bacterium *Buchnera*. Zoolog. Sci..

[87] Stoll S., Feldhaar H., Fraunholz M.J., Gross R. (2010). Bacteriocyte dynamics during development of a holometabolous insect, the carpenter ant *Camponotus floridanus*. BMC Microbiol..

[88] Stoll S., Feldhaar H., Gross R. (2008). Transcriptional profiling of the endosymbiont *Blochmannia floridanus* during different developmental stages of its holometabolous ant host. Environ. Microbiol..

[89] Feldhaar H., Straka J., Krischke M., Berthold K., Stoll S., Mueller M.J., Gross R. (2007). Nutritional upgrading for omnivorous carpenter ants by the endosymbiont *Blochmannia*. BMC Biol..

[90] Login F.H., Balmand S., Vallier A., Vincent-Monegat C., Vigneron A., Weiss-Gayet M., Rochat D., Heddi A. (2011). Antimicrobial peptides keep insect endosymbionts under control. Science.

[91] Heddi A., Charles H., Khatchadourian C., Bonnot G., Nardon P. (1998). Molecular characterization of the principal symbiotic bacteria of the weevil *Sitophilus oryzae*: A peculiar G + C content of an endocytobiotic DNA. J. Mol. Evol..

[92] Chapman E., Farr G.W., Usaite R., Furtak K., Fenton W.A., Chaudhuri T.K., Hondorp E.R., Matthews R.G., Wolf S.G., Yates J.R. et al. (2006). Global aggregation of newly translated proteins in an *Escherichia coli* strain deficient of the chaperonin GroEL. Proc. Natl. Acad. Sci. USA.

[93] Haine E.R. (2008). Symbiont-mediated protection. Proc. R. Soc. Lond B Biol. Sci..

[94] Feldhaar H. (2011). Bacterial symbionts as mediators of ecologically important traits of insect hosts. Ecol. Entomol..

[95] Oliver K.M., Russell J.A., Moran N.A., Hunter M.S. (2003). Facultative bacterial symbionts in aphids confer resistance to parasitic wasps. Proc. Natl. Acad. Sci. USA.

[96] Vorburger C., Gehrer L., Rodriguez P. (2010). A strain of the bacterial symbiont *Regiella insecticola* protects aphids against parasitoids. Biol. Lett..

[97] von Burg S., Ferrari J., Muller C.B., Vorburger C. (2008). Genetic variation and covariation of susceptibility to parasitoids in the aphid *Myzus persicae*: No evidence for trade-offs. Proc. R. Soc. Lond B Biol. Sci..

[98] Ferrari J., Darby A.C., Daniell T.J., Godfray H.C.J., Douglas A.E. (2004). Linking the bacterial community in pea aphids with host-plant use and natural enemy resistance. Ecol. Entomol..

[99] Angalet G.W., Fuester R. (1977). Aphidius Hymenoptera-Aphididiidae parasites of pea aphid *Acyrthosiphon pisum* Homoptera-Aphididae in Eastern Half of United-States Homoptera-Aphididae. Ann. Entomol. Soc. Am..

[100] Oliver K.M., Moran N.A., Hunter M.S. (2006). Costs and benefits of a superinfection of facultative symbionts in aphids. Proc. R. Soc. Lond B Biol. Sci..

[101] Oliver K.M., Campos J., Moran N.A., Hunter M.S. (2008). Population dynamics of defensive symbionts in aphids. Proc. R. Soc. Lond B Biol. Sci..

[102] Degnan P.H., Moran N.A. (2008). Diverse phage-encoded toxins in a protective insect endosymbiont. Appl. Environ. Microbiol..

[103] Moran N.A., Degnan P.H., Santos S.R., Dunbar H.E., Ochman H. (2005). The players in a mutualistic symbiosis: Insects, bacteria, viruses, and virulence genes. Proc. Natl. Acad. Sci. USA.

[104] Scarborough C.L., Ferrari J., Godfray H.C. (2005). Aphid protected from pathogen by endosymbiont. Science.

[105] Xie J., Vilchez I., Mateos M. (2010). *Spiroplasma* bacteria enhance survival of *Drosophila hydei* attacked by the parasitic wasp *Leptopilina heterotoma*. PLoS One.

[106] Jaenike J., Unckless R., Cockburn S.N., Boelio L.M., Perlman S.J. (2010). Adaptation via symbiosis: Recent spread of a *Drosophila* defensive symbiont. Science.

[107] Jaenike J., Brekke T.D. (2011). Defensive endosymbionts: A cryptic trophic level in community ecology. Ecol. Lett..

[108] Kaltenpoth M., Schmitt T., Polidori C., Koedam D., Strohm E. (2010). Symbiotic streptomycetes in antennal glands of the South American digger wasp genus *Trachypus* (Hymenoptera, Crabronidae). Physiol. Entomol..

[109] Currie C.R., Scott J.A., Summerbell R.C., Malloch D. (1999). Fungus-growing ants use antibiotic-producing bacteria to control garden parasites. Nature.

[110] Scott J.J., Oh D.C., Yuceer M.C., Klepzig K.D., Clardy J., Currie C.R. (2008). Bacterial protection of beetle-fungus mutualism. Science.

[111] Kellner R.L.L., Dettner K. (1996). Differential efficacy of toxic pederin in deterring potential arthropod predators of *Paederus *(Coleoptera: Staphylinidae) offspring. Oecologia.

[112] Kellner R.L.L. (2003). Stadium-specific transmission of endosymbionts needed for pederin biosynthesis in three species of *Paederus* rove beetles. Entomol. Exp. Appl..

[113] Piel J., Hofer I., Hui D.Q. (2004). Evidence for a symbiosis island involved in horizontal acquisition of pederin biosynthetic capabilities by the bacterial symbiont of *Paederus fuscipes* beetles. J. Bacteriol..

[114] Hoffmann A.A., Hercus M., Dagher H. (1998). Population dynamics of the *Wolbachia* infection causing cytoplasmic incompatibility in *Drosophila melanogaster*. Genetics.

[115] Hedges L.M., Brownlie J.C., O’Neill S.L., Johnson K.N. (2008). *Wolbachia* and virus protection in insects. Science.

[116] Glaser R.L., Meola M.A. (2010). The native *Wolbachia* endosymbionts of *Drosophila melanogaster* and *Culex quinquefasciatus* increase host resistance to West Nile virus infection. PLoS One.

[117] Kambris Z., Cook P.E., Phuc H.K., Sinkins S.P. (2009). Immune activation by life-shortening *Wolbachia* and reduced filarial competence in mosquitoes. Science.

[118] Wachinger M., Kleinschmidt A., Winder D., von Pechmann N., Ludvigsen A., Neumann M., Holle R., Salmons B., Erfle V., Brack-Werner R. (1998). Antimicrobial peptides melittin and cecropin inhibit replication of human immunodeficiency virus 1 by suppressing viral gene expression. J. Gen. Virol..

[119] Carballar-Lejarazu R., Rodriguez M.H., de la Cruz Hernandez-Hernandez F., Ramos-Castaneda J., Possani L.D., Zurita-Ortega M., Reynaud-Garza E., Hernandez-Rivas R., Loukeris T., Lycett G. et al. (2008). Recombinant scorpine: A multifunctional antimicrobial peptide with activity against different pathogens. Cell Mol. Life Sci..

[120] Gwadz R.W., Kaslow D., Lee J.Y., Maloy W.L., Zasloff M., Miller L.H. (1989). Effects of magainins and cecropins on the sporogonic development of malaria parasites in mosquitoes. Infect Immun..

[121] Kim W., Koo H., Richman A.M., Seeley D., Vizioli J., Klocko A.D., O’Brochta D.A. (2004). Ectopic expression of a cecropin transgene in the human malaria vector mosquito *Anopheles gambiae* (Diptera: Culicidae): Effects on susceptibility to *Plasmodium*. J. Med. Entomol..

[122] Povelones M., Waterhouse R.M., Kafatos F.C., Christophides G.K. (2009). Leucine-rich repeat protein complex activates mosquito complement in defense against *Plasmodium* parasites. Science.

[123] Weiss B.L., Wang J., Aksoy S. (2011). Tsetse immune system maturation requires the presence of obligate symbionts in larvae. PLoS Biol..

[124] Weiss B.L., Maltz M., Aksoy S. (2012). Obligate symbionts activate immune system development in the tsetse fly. J. Immunol..

[125] Aksoy S. (2000). Tsetse-A haven for microorganisms. Parasitol. Today..

[126] Attardo G.M., Lohs C., Heddi A., Alam U.H., Yildirim S., Aksoy S. (2008). Analysis of milk gland structure and function in *Glossina morsitans*: Milk protein production, symbiont populations and fecundity. J. Insect Physiol..

[127] Nogge G. (1981). Significance of symbionts for the maintenance of an optimal nutritional state for successful reproduction in hematophagous arthropods. Parasitology.

[128] Rizki T.M., Rizki R.M., Grell E.H. (1980). A mutant affecting the crystal cells in *Drosophila melanogaster*. Roux Arch. Dev. Biol..

[129] Blandin S.A., Levashina E.A. (2007). Phagocytosis in mosquito immune responses. Immunol. Rev..

[130] Lebestky T., Chang T., Hartenstein V., Banerjee U. (2000). Specification of *Drosophila* hematopoietic lineage by conserved transcription factors. Science.

[131] Haine E.R., Moret Y., Siva-Jothy M.T., Rolff J. (2008). Antimicrobial defense and persistent infection in insects. Science.

[132] Gross R., Vavre F., Heddi A., Hurst G.D., Zchori-Fein E., Bourtzis K. (2009). Immunity and symbiosis. Mol. Microbiol..

